# Expanding the clinical phenotype and genetic spectrum of GEMIN5 disorders: Early‐infantile developmental and epileptic encephalopathies

**DOI:** 10.1002/brb3.3535

**Published:** 2024-05-21

**Authors:** Jing Zhang, Xinting Liu, Gang Zhu, Lin Wan, Yan Liang, Nannan Li, Mingwei Huang, Guang Yang

**Affiliations:** ^1^ Senior Department of Pediatrics, The Seventh Medical Center of Chinese PLA General Hospital Beijing China; ^2^ Department of Pediatrics, The First Medical Center of Chinese PLA General Hospital Beijing China; ^3^ Medical School of Chinese People's Liberation Army Beijing China; ^4^ Aegicare (Shenzhen) Technology Co., Ltd Shenzhen China; ^5^ The Second School of Clinical Medicine Southern Medical University Guangzhou China

**Keywords:** early‐infantile developmental and epileptic encephalopathies, epilepsy, GEMIN5 gene, neurodevelopmental disorder with cerebellar atrophy and motor dysfunction syndrome, whole‐exome sequencing

## Abstract

**Background:**

Several biallelic truncating and missense variants of the gem nuclear organelle–associated protein 5 (*GEMIN5*) gene have been reported to cause neurodevelopmental disorders characterized by cerebellar atrophy, intellectual disability, and motor dysfunction. However, the association between biallelic *GEMIN5* variants and early‐infantile developmental and epileptic encephalopathies (EIDEEs) has not been reported.

**Purpose:**

This study aimed to expand the phenotypic spectrum of *GEMIN5* and explore the correlations between epilepsy and molecular sub‐regional locations.

**Methods:**

We performed whole‐exome sequencing in two patients with EIDEE with unexplained etiologies. The damaging effects of variants were predicted using multiple in silico tools and modeling. All reported patients with *GEMIN5* pathogenic variants and detailed neurological phenotypes were analyzed to evaluate the genotype–phenotype relationship.

**Results:**

Novel biallelic *GEMIN5* variants were identified in two unrelated female patients with EIDEE, including a frameshift variant (Hg19, chr5:154284147‐154284148delCT: NM_015465: c.2551_c.2552delCT: p.(Leu851fs*30)), a nonsense mutation (Hg19, chr5:154299603‐154299603delTinsAGA: NM_015465: c.1523delTinsAGA: p.(Leu508*)), and two missense variants (Hg19, chr5:154282663T > A: NM_015465: c.2705T > A: p.(Leu902Gln) and Hg19, chr5:154281002C > G: NM_015465: c.2911C > G: p.(Gln971Glu)), which were inherited from asymptomatic parents and predicted to be damaging or probably damaging using in silico tools. Except p.Leu508*, all these mutations are located in tetratricopeptide repeat (TPR) domain. Our two female patients presented with seizures less than 1 month after birth, followed by clusters of spasms. Brain magnetic resonance imaging suggests dysgenesis of the corpus callosum and cerebellar hypoplasia. Video electroencephalogram showed suppression‐bursts. Through a literature review, we found 5 published papers reporting 48 patients with biallelic variants in *GEMIN5*. Eight of 48 patients have epilepsy, and 5 patients started before 1 year old, which reminds us of the relevance between *GEMIN5* variants and EIDEE. Further analysis of the 49 *GEMIN5* variants in those 50 patients demonstrated that variants in TPR‐like domain or RBS domain were more likely to be associated with epilepsy.

**Conclusions:**

We found novel biallelic variants of *GEMIN5* in two individuals with EIDEE and expanded the clinical phenotypes of *GEMIN5* variants. It is suggested that the *GEMIN5* gene should be added to the EIDEE gene panel to aid in the clinical diagnosis of EIDEE and to help determine patient prognosis.

## 1 INTRODUCTION

*GEMIN5* gene (known as GEM‐ASSOCIATED PROTEIN 5 in Online Mendelian Inheritance in Man [OMIM], MIM#607005), mapped to 5q33.2, encodes gem nuclear organelle–associated protein 5 (GEMIN5) (Gubitz et al., 2002). GEMIN5 is a WD repeat protein and a component of the survival of motor neurons (SMN) complex. GEMIN5 contains different structural domains, including the WD40 repeat domain located at the N‐terminal region (1‐739AA), a tetratricopeptide repeat (TPR)‐like dimerization domain with 17 helices (845‐1097AA), and a bipartite non‐conventional RNA‐binding site (designated as RBS1 and RBS2, 1287‐1508AA) (Embarc‐Buh et al., 2021; Jin et al., 2016; Moreno‐Morcillo et al., 2020). GEMIN5 is involved in the spliceosomal small nuclear ribonucleoprotein biogenesis (Francisco‐Velilla et al., 2022), acting as a signal recognition particle‐interacting protein (Piazzon et al., 2013), ribosome interacting factor (Francisco‐Velilla et al., 2016), and mRNA translation regulator (Martinez‐Salas et al., 2020). WD40 repeat domain is involved in the recognition of snRNAs (Francisco‐Velilla et al., 2022). TRP‐like domain with 17 helices that oligomerizes as a canoe‐shaped homodimer is vital for protein architecture and activity (Ibrahim et al., 2023). RBS1–RBS2 exerts multiple cellular functions such as RNA‐binding specificity and affinity, as well as translation repression and control of the protein stability (Embarc‐Buh et al., 2021; Jin et al., 2016; Moreno‐Morcillo et al., 2020).

Clinically, variants in *GEMIN5* have been associated with neurodevelopmental disorder with cerebellar atrophy and motor dysfunction (NEDCAM) syndrome (OMIM#619333) (Ibrahim et al., 2023), neurodevelopmental disorder characterized by cerebellar atrophy, developmental and cognitive delay, ataxia, motor dysfunction, and hypotonia. The association between *GEMIN5* variants and epilepsy has not been fully understood and given enough attention. Here, we reported two patients harboring biallelic variants in the *GEMIN5* gene and suffering from early‐infantile developmental and epileptic encephalopathies (EIDEE) (Zuberi et al., 2022), but otherwise presenting with clinical features distinct from NEDCAM syndrome, broadening the phenotypic spectrum of *GEMIN5*. We also analyzed all 48 patients with *GEMIN5* variants, focusing on the correlations between epilepsy and molecular sub‐regional locations.

## 2 MATERIALS AND METHODS

### 2.1 Subjects

Patients with unexplained EIDEE were recruited from the First Medical Center of Chinese PLA General Hospital between July 2020 and May 2023. Written informed consent was obtained from the patients and their parents. The project was approved by the Ethical Committee of the Chinese PLA General Hospital. All the study procedures were performed in accordance with the Declaration of Helsinki. Detailed clinical information was collected, such as age, gender, seizure types and frequencies, general and neurological examination results, family history, responses to anti‐seizure medicines (ASMs), results of video‐electroencephalography (VEEG), and cranial magnetic resonance imaging (MRI). EIDEE or epilepsies were diagnosed according to the criteria of the Commission on Classification and Terminology of the ILAE (Zuberi et al., 2022). EIDEE includes Ohtahara syndrome and early myoclonic encephalopathy, previously categorized as neonatal and infantile epileptic syndrome (Lombroso, 1990; Yelin et al., 1999). EIDEE is a syndrome characterized by seizures in the first 3 months of life, frequent seizures, drug resistance, and developmental delay. Patients have abnormal interictal electroencephalograms that may include suppression‐bursts (SBs), diffuse slow waves, or multifocal discharges (Ohtahara & Yamatogi, 2006). Neuroimaging, metabolic, and genetic testing provide a precise etiologic diagnosis in approximately 80% of patients (Bayat et al., 2021).

### 2.2 Trio‐based whole‐exome sequencing (WES)

Venous blood was taken from consenting patients and their parents by EDTA anticoagulant tube. Then genomic DNA was extracted from venous blood samples using a RelaxGene Blood DNA system (Tiangen Biotech Co., Ltd.). Then the libraries for whole‐exome sequencing were constructed with NanoPrep DNA Library Preparation Module (for MGI), 96 rxn. The libraries were then sequenced on a BGI MGISEQ‐2000 sequencer. After obtaining the raw reads, read alignment was performed using the Burrows‐Wheeler Aligner tool (version 0.7.17) with default parameters against the human genome assembly hg19 (GRCh37) as previously described (Li & Durbin, 2010). The generated bam file was then sorted and deduplicated by SAMtools (Li et al., 2009) and Picard, respectively. Then Genome Analysis Toolkit (GATK; https://software.broadinstitute.org/gatk/) was applied to detect SNVs and indels (<50 bp), and CNVkit was performed to detect the copy number variations (Talevich et al., 2016). The 1000 Genome Project, Genome Aggregation Database, Exome Aggregation Consortium, and others were employed to annotate the variant frequency in the general population. In addition, the OMIM and Human Gene Mutation Database (HGMD) and ClinVar (NIH Clinical Genomic Resource) were employed to annotate the related diseases.

### 2.3 Mutation analysis

We aimed to evaluate the relationship between the genotype and phenotype through exhaustively searching *GEMIN5* pathogenic variants on PubMed up until May 2023 to identify studies published in English using the following terms: *GEMIN5*, epilepsy, seizure, EIDEE, and NEDCAM syndrome. All pathogenic variants in patients with neurological phenotypes were included and analyzed. We performed molecular modeling analysis of the variants in protein structure. Pymol was used for protein structure mapping. The species conservation analysis of GEMIN5 protein has been conducted.

## 3 RESULTS

### 3.1 Identification of *GEMIN5* variants

**FIGURE 1:**
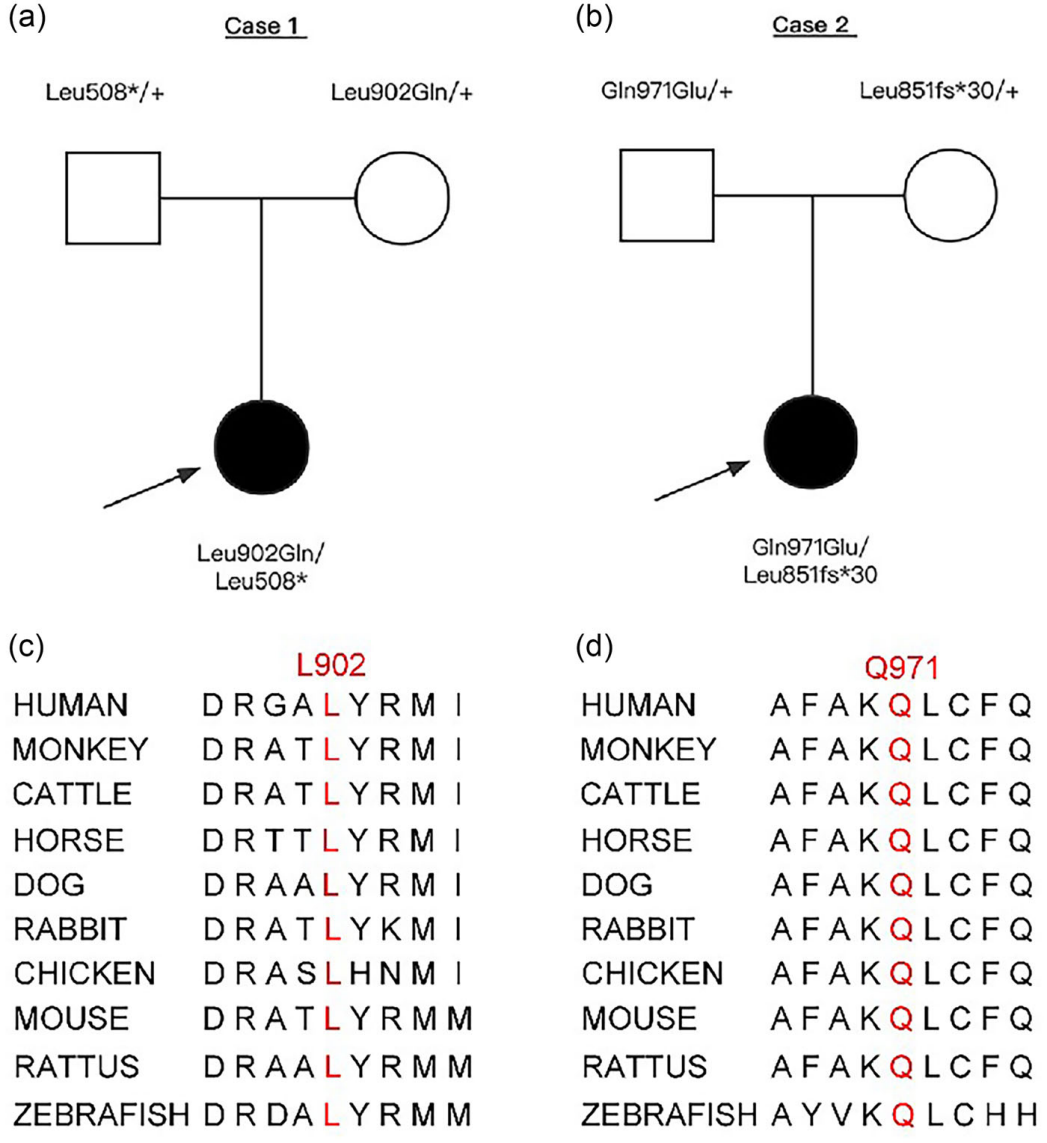
Genetic data on the patients with gem nuclear organelle–associated protein 5 (*GEMIN5*) variants. (a and b) Pedigree of the two families. The black arrow points to the probands. (c and d) The variant amino acids in our patients were conserved from multiple species.

Two patients with biallelic truncating and missense variants in *GEMIN5* were identified (Hg19, chr5:154282663T > A: NM_015465: c.2705T > A: p.(Leu902Gln) and Hg19, chr5:154299603‐154299603delTinsAGA: NM_015465: c.1523delTinsAGA: p.(Leu508*) for patient 1; Hg19, chr5:154281002C > G: NM_015465: c.2911C > G: p.(Gln971Glu) and Hg19, chr5:154284147‐154284148delCT: NM_015465: c.2551_c.2552delCT: p.(Leu851fs*30) for patient 2) (Table 1; Figure 1a,b). Two patients had no other pathogenic or likely pathogenic variants. Variants of *GEMIN5* were annotated based on transcript NM_015465, and the missense variants of two patients were confirmed by Sanger sequencing.

**TABLE 1:** Epileptic patients with gem nuclear organelle–associated protein 5 (GEMIN5) variants.

References	Present study Patient 1		Present study Patient 2		Kour et al. (2021 Patient 18		Kour et al. (2021) Patient 20		Kour et al. (2021) Patient 26		Ibrahm et al. Patient II.4	Kour et al. (2021) Patient 4	Kour et al. (2021) Patient 17		Kour et al. (2021) Patient 24		Kour et al. (2021) Patient 27	
Age at last observation	20 months		17 months		8 years		7 years		18 months		28 years	2 years	15 years		29 years		31 months	
Sex	F		F		F		M		F		M	M	M		M		F	
cDNA change/Proteinalteration	c.2705T > A/p.Leu902Gln	c.1523delTinsAGA/p.Leu508*	c.2911C > G/p.Gln971Glu	c.2551_c.2552delCT/p.Leu851fs*30	c.4100T > C/p.Leu1367Pro	c.3057C > A/p.Asp1019Glu	c.3844 T > C/p.Tyr1282His	c.217T > C/p.Ser73Pro	c.485A > G/p.His162Arg	c.4100T > C/p.Leu1367Pro	c.3162_3164delHomozygous/Asp1054_Ala1055delinsGlu	c.2768A > CHomozygous/p.His923Pro	c.2962A > T/p.lle988Phe	c.4100T > C/p.Leu1367Pro	c.2962 A > T/p.Ile988Phe	c.3930_3933delCTCT/p.Ser1311LeufsTer7	c.282G > A/p.Trp94Ter	c.3856T > A/p.Tyr1286Asn
Inheritance	Maternal	Paternal	Paternal	Maternal	Paternal	Maternal	Paternal	Maternal	Maternal	Paternal	Maternal/Paternal	Maternal/Paternal	Paternal	Maternal	Paternal	Maternal	Paternal	Maternal
Variant type	Missense mutation	Nonsense mutation	Missense mutation	Frameshift mutation	Missense mutation	Missense mutation	Missense mutation	Missense mutation	Missense mutation	Missense mutation	Frameshift mutation	Missense mutation	Missense mutation	Missense mutation	Missense mutation	Frameshift mutation	Missense mutation	Missense mutation
Domain	TPR‐like	WD40	TPR‐like	TPR‐like	RBS1	TPR‐like	Between TPR‐like and RBS1	WD40	WD40	RBS1	TPR‐like	TPR‐like	TPR‐like	RBS1	TPR‐like	RBS1	WD40	Between TPR‐like and RBS1
Birth parameters	Normal			36 weeks and 5 days preterm infant; Congenital chondrodysplasia of the larynx; Congenital clubfoot on the right side; Bilateral hip dislocation		NA		NA		NA		NA	Severe hypotonia requiring resuscitation at birth; a weak cry with no visual tracking, severe hypotonia, absence of anti‐gravity movements, and areflexia	NA		NA		NA
Developmental milestones/Follow up	No motor or language development until 20 months old		Hold her head up and make unconscious pronunciation at 17 months old		NA		NA		NA		NA	NA	NA		NA		Cannot sit or stand but can roll over; Can verbalize repetitive syllables but not words (at 31 months old)	
Onset of seizures	20 days		7 days		6 years		Neonatal		Neonatal		<1 year	Neonatal	NA		5–6 years old		3 weeks	
Epilepsy syndrome	EIDEE		EIDEE		SeLECTS		DEE		NA		NA	NA	NA		NA		NA	
Seizure types	Focal motor seizure; Epileptic spasms; GTCS		Tonic seizures; GTCS; Epileptic spasm		NA		NA		GTCS		NA	NA	NA		Generalized seizures		NA	

References	Present studyPatient 1	Present studyPatient 2	Kour et al. (2021)Patient 18	Kour et al. (2021)Patient 20	Kour et al. (2021)Patient 26	Ibrahm et al.Patient II.4	Kour et al. (2021)Patient 4	Kour et al. (2021)Patient 17	Kour et al. (2021)Patient 24	Kour et al. (2021)Patient 27
ASM	VPA; TPM; LEV; VGB; PB; LGT; CLB; ZNS	TPM; VPA; ZNS; LGT; CLB; VGB	NA	NA	CLB	NA	NA	NA	NA	LEV
Outcome of epilepsy	Refractory	Refractory	Controlled	Refractory	Controlled	NA	Refractory (dead at 3 years old)	Refractory	Controlled	Controlled
EEG	SBs	SBs	Centrotemporal spikes	1. Mild diffuse background slowing 2. Spikes, bilateral centrotemporal, frequent	Abnormal	Seizure	Seizure	NA	An abnormal EEG during awakeness and sleep stages due to background mildly diffused slowing. This is consistent with mild cerebral dysfunction. No clinical or electrographic seizures were recorded	NA
MRI (age)	Dysgenesis of the corpus callosum and cerebellar hypoplasia (approximately 6 months old)	Dysgenesis of the corpus callosum and cerebellar hypoplasia (approximately 6 months old)	Moderate‐to‐severe cerebellar volume loss	A progressive cerebellar atrophy with mild cerebellar cortex T2 hyperintensity	Cerebellar vermian and hemispheric atrophy	Mild cerebral atrophy	Vermis predominant cerebellar atrophy that was also not before (6 months)	Cerebellar atrophy along with volume loss in the pons/brainstem	Cerebellar atrophy Progression of cerebellar volume loss	Cerebellar atrophy and hyperintensities of the cerebellar cortex (at 10 months old); Progressive cerebellar atrophy with cerebellar cortex hyperintensities (at the 2½ years later)
Development	Y	Y	Y	Y	Y	Y	Y	Y	Y	Y
Delayed Regression	N	N	N	N	N	N	N	N	N	N
Moter delayed	Y	Y	Y	Y	Y	Y	Y	Y	Y	Y
Speech delayed	Y	Y	Y	Y	N	Y	Y	Y	Y	Y
Cognitive delayed	Y	Y	Y (mild)	Y	N	Y	Y	Y	Y	Y

Abbreviations: ASM, anti‐seizure medicine; CLB, clobazam; DEEs, developmental and epileptic encephalopathies; EEG, electroencephalography; EIDEE, early‐infantile developmental and epileptic encephalopathies; GTCS, generalized tonic–clonic seizure; LEV, levetiracetam; LGT, lamotrigine; MRI, magnetic resonance imaging; NA, not applicable; PB, phenobarbital; SBs, suppression‐bursts; SeLECTS, self‐limited childhood epilepsy with centrotemporal spikes; TPM, topiramate; VGB, vigabatrin; VPA, sodium valproate; ZNS, zonisamide.

The amino acid residues of the two missense variants are highly conserved in various species (Figure 1c,d). The two missense variants were suggested to be damaging or probably damaging by four tools (Table 2). The pathogenicity of p.Leu902Gln and p.Gln971Glu was predicted as uncertain, whereas p. Leu508* and p. Leu851fs*30 were predicted as likely pathogenic, according to ACMG code‐level (Table 2).

**TABLE 2:** Genetic features of the individuals with gem nuclear organelle–associated protein 5 (GEMIN5) variants.

Patients	cDNA change/Protein alteration/Transcripts	MAF	Variant class	Inheritance	ACMG code‐level	SIFT	Polyphen2 HDIV Pred	Polyphen2 HVAR Pred	Mutation Taster Pred
1	Hg19, chr5:154282663T > A: NM_015465: c.2705T > A: p.(Leu902Gln)	–	Missense mutation	Maternal	Uncertain PM2+PM3_ Supporting	D (0)	PD (0.986)	PD (0.965)	D (Gubitz et al., 2002)
	Hg19, chr5:154299603‐154299603delTinsAGA: NM_015465: c.1523delTinsAGA: p.(Leu508*)	–	Nonsense mutation	paternal	Likely pathogenic PVS1+PM2_ Supporting	–	–	–	–
2	Hg19, chr5:154281002C > G: NM_015465: c.2911C > G: p.(Gln971Glu)	–	Missense mutation	paternal	Uncertain PM2+PM3+PP3_ Supporting	D (0.005)	PD (Gubitz et al., 2002)	PD (0.998)	D (0.991089)
	Hg19, chr5:154284147‐154284148delCT: NM_015465: c.2551_c.2552delCT: p.(Leu851fs*30)	–	frameshift variant	maternal	Likely pathogenic PVS1+PM2_ Supporting	–	–	–	–

Abbreviations: D, damaging; MAF, minor allele frequency from Genome Aggregation Database; NA, not applicable; PD, probably damaging; PM2_Supporting, absent from controls in Exome Sequencing Project, 1000 Genomes Project, or Exome Aggregation Consortium; PM3_Supporting, for recessive disorders, detected in trans with a pathogenic variant; PP3_Supporting, multiple lines of computational evidence support a deleterious effect on the gene or gene product; PVS1_Supporting, the mutation causes changes in protein function.

### 3.2 Clinical information

The main clinical features of the two patients are summarized in Table 1. Two patients were both born to non‐consanguineous parents.

**FIGURE 2:**
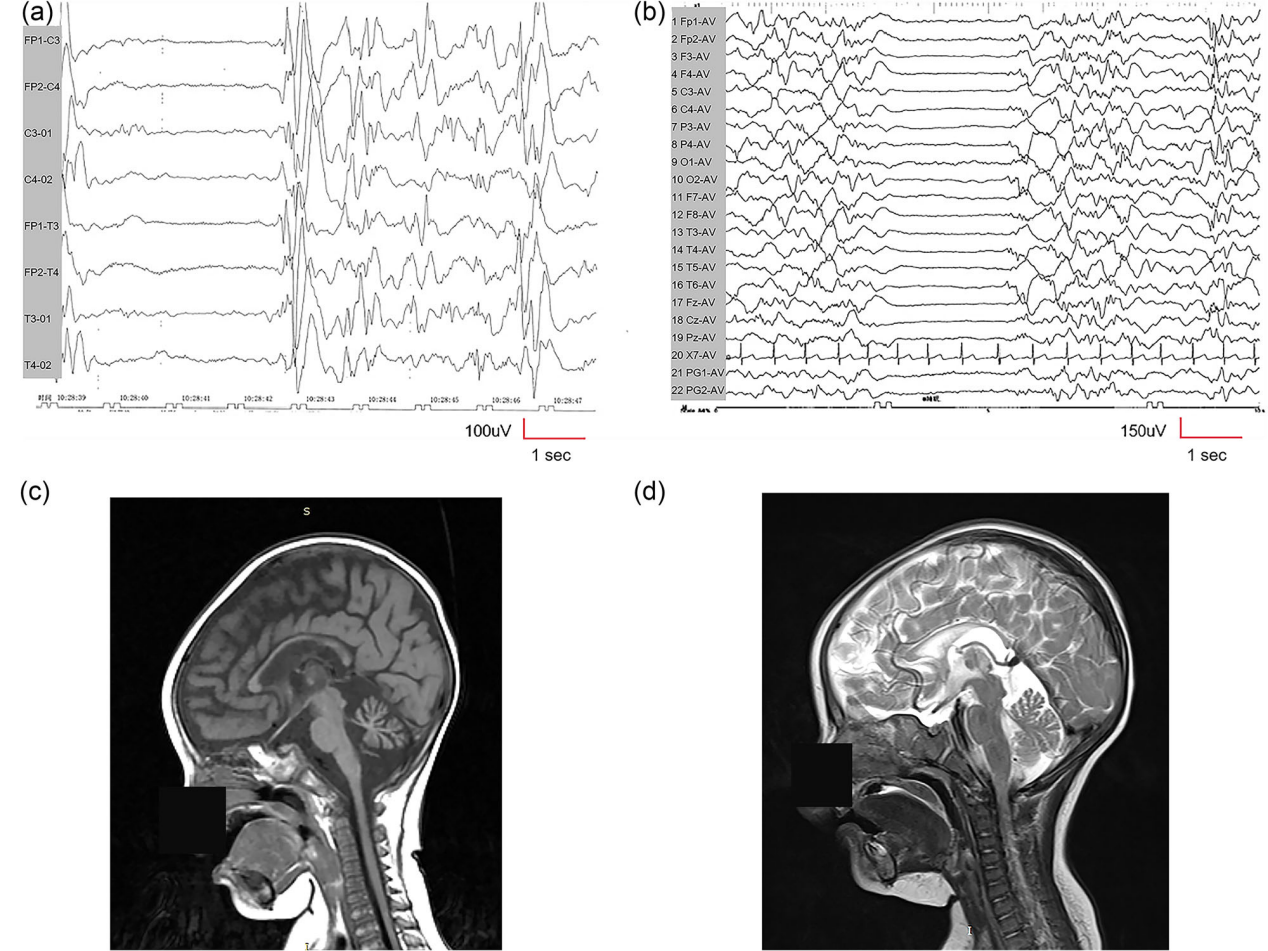
Electroencephalogram (EEG) and neuroimaging results of the patients with gem nuclear organelle–associated protein 5 (*GEMIN5*) variants. (a) Patient 1, female, approximately 1 month old. EEG during awake without anti‐seizure medicine (ASM) (bipolar longitudinal montage. Sensitivity 100 uV/cm, high‐pass filter 1 Hz, low‐pass filter 70 Hz): There are suppression‐bursts (SBs). (b) Patient 2, female, approximately 6 months old. EEG during awakeness with the use of levetiracetam (LEV), topiramate (TPM), vigabatrin (VGB), and clobazam (CLB) (average montage. Sensitivity 150 uV/cm, high pass filter 1 Hz, low pass filter 70 Hz): There are SBs. (c) Cranial magnetic resonance imaging (MRI) of patient 1 (approximately 6 months old) showed dysgenesis of the corpus callosum and cerebellar hypoplasia in sagittal T1. (d) Cranial MRI of patient 2 (approximately 6 months old) showed dysgenesis of the corpus callosum and cerebellar hypoplasia in sagittal T2.

Patient 1, a 20‐month‐old girl, is the first child of healthy parents without any family history of intellectual disability (ID) or epilepsy (Figure 1a). She was delivered by full‐term cesarean section after an uneventful pregnancy. The patient experienced focal motor seizure 20 days after birth. The seizure types later changed to clusters of epileptic spasms and generalized tonic–clonic seizures (GTCSs). Neurological examination revealed hypotonia. VEEG showed SBs at approximately 1 month old (Figure 2a). Cranial MRI suggests dysgenesis of the corpus callosum and cerebellar hypoplasia at approximately 6 months old (Figure 2c). Blood amino acid and urinary organic acid screening results did not show significant abnormalities. She was treated sequentially with corticotropin (ACTH), oral glucocorticoids, sodium valproate (VPA), topiramate (TPM), levetiracetam (LEV), vigabatrin (VGB), phenobarbital (PB), ketogenic diet, lamotrigine (LGT), clobazam (CLB), and zonisamide (ZNS), but the seizures were not effectively controlled at 6 months old (Table 1). Subsequently, a diagnosis of EIDEE was made based on her clinical and EEG features. Her psychomotor development was behind that of healthy children of the same age at 6 months old, and she did not exhibit vocalizations or cooing, hold her head up, or follow voice and vision. Through telephone follow‐up, patient 1 experienced multiple isolated epileptic spasms daily at 20 months old. Patient 1 still lacks head control, rolling over, sitting independently, or vocalization. Patient 1 is currently taking CLB and ZNS, with caregivers reporting a relatively positive response to CLB. No follow‐up MRI was conducted post‐discharge, and the repeat VEEG showed similar findings as before.

Patient 2, a 17‐month‐old girl, is the first child of unrelated, healthy parents without any family history of ID or epilepsy (Figure 1b). Her mother was diagnosed with hypertension 3 days before delivery, but the details were not known. Patient 2 was delivered at 36 + 5 weeks with meconium‐stained amniotic fluid, a knotted umbilical cord, and no hypoxic asphyxia at birth. She developed tonic seizures with no obvious cause 1 week after birth. She had experienced GTCS 1 month later, which lasted for about 2 min and then resolved on its own. The seizure types later changed to clusters of epileptic spasm. Neurological examination revealed hypertonia. She also has congenital chondrodysplasia of the larynx, congenital clubfoot on the right side, and bilateral hip dislocation. VEEG during the interictal period showed SBs at approximately 6 months old (Figure 2b). Cranial MRI suggests dysgenesis of the corpus callosum and cerebellar hypoplasia at approximately 6 months old (Figure 2d). She was treated sequentially with ACTH, oral glucocorticoids, TPM, VPA, ZNS, CLB, and VGB, and the seizures were not effectively controlled (Table 1). Her growth and development were behind that of healthy children of the same age at 6 months old, and she did not exhibit vocalizations or cooing, hold her head up, or follow voice and vision. Through telephone follow‐up, patient 2 experienced four to five episodes of spasms in the upper limbs per day at 17 months old, with spontaneous recovery within a few seconds. Patient 2 can maintain head control for approximately 5 min, cannot sit independently or roll over, but is able to vocalize unconsciously. The patient is currently taking VPA, LGT, and CLB, with caregivers reporting the best seizure control with CLB. No follow‐up MRI was conducted post‐discharge, and the repeat VEEG showed similar findings as before.

### 3.3 Structure alteration of GEMIN5 protein

**FIGURE 3:**
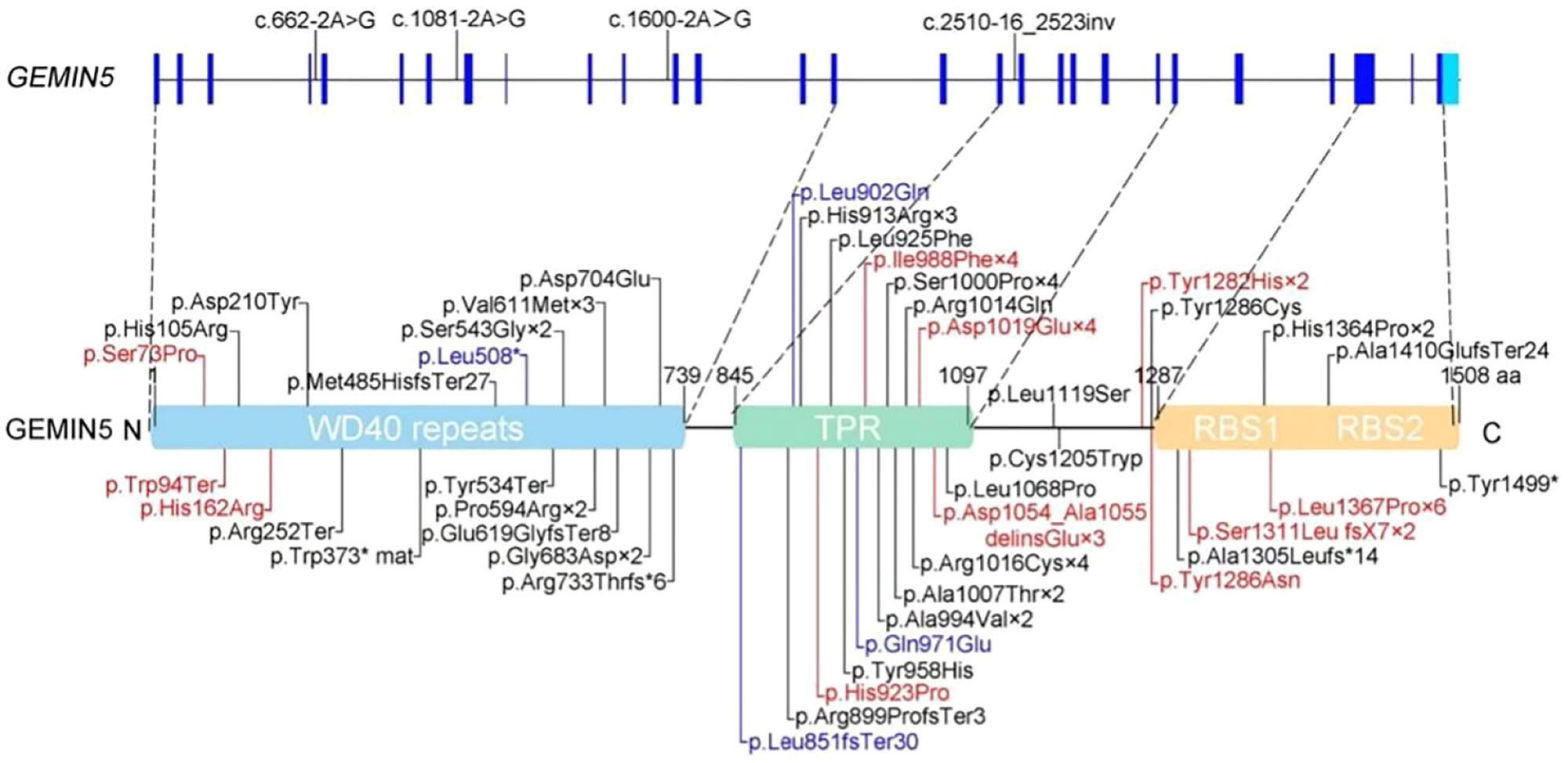
Schematic diagram of gem nuclear organelle–associated protein 5 (*GEMIN5*) and the localization of the variants of *GEMIN5* identified in previous reports (variants that cause epilepsy are highlighted in red and variants that do not cause epilepsy are highlighted in black) and in our study (highlighted in blue). The number represents the number of patients with this mutation.

**FIGURE 4:**
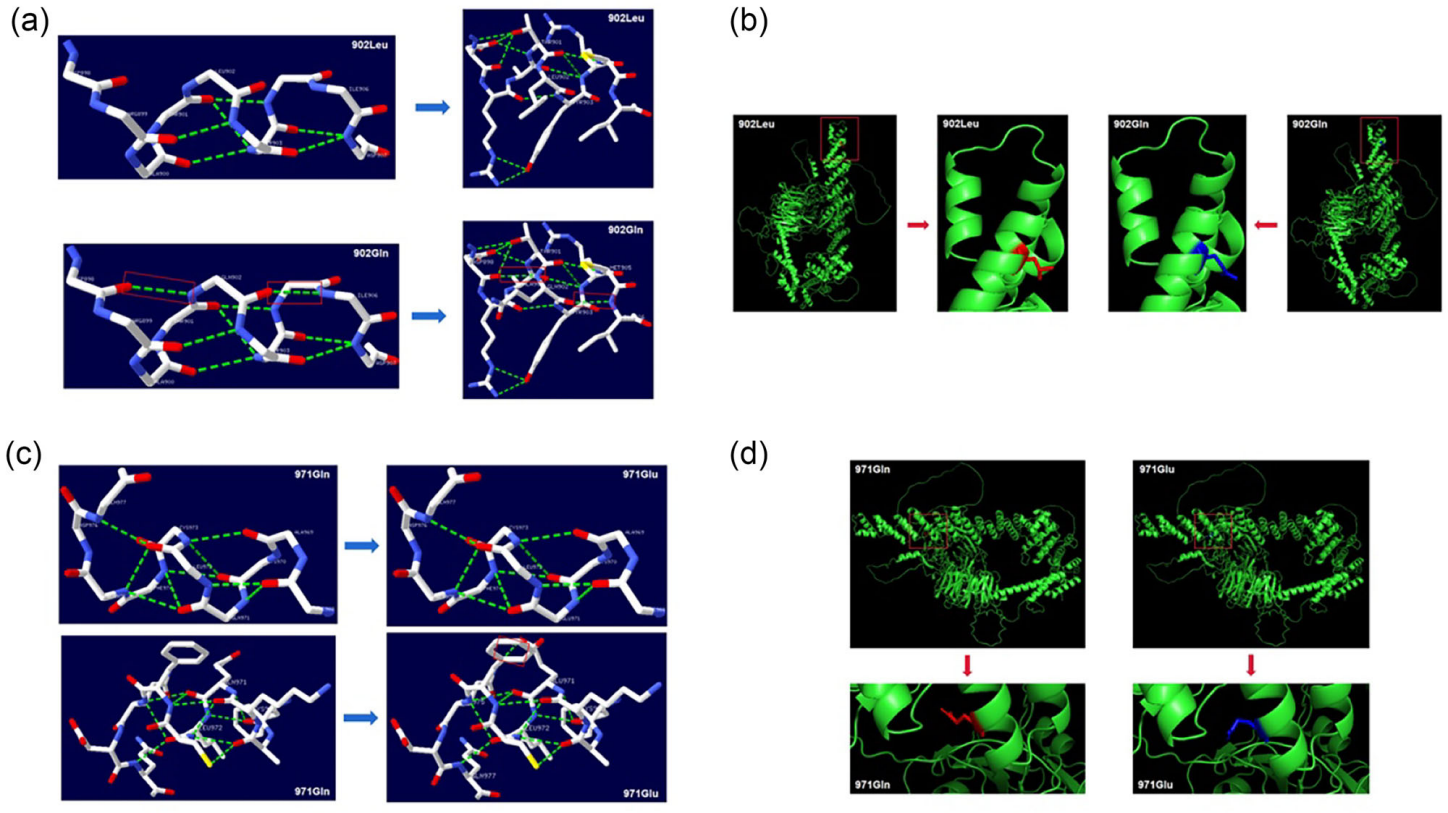
Changes in gem nuclear organelle–associated protein 5 (GEMIN5) structure. (a and b) The protein changes of Leu902Gln. (c and d) The protein changes of Gln971Glu. In (a) and (c), white represents carbon atoms, red represents oxygen atoms, yellow represents sulfur atoms, blue represents nitrogen atoms, and the green dotted lines represent hydrogen bonds.

As shown in Figure 3, GEMIN5 contains the WD40 repeat domain, TPR‐like domain, RBS1, and RBS2. Structural model of GEMIN5 indicated variants p.Leu902Gln, p.Gln971Glu, and p.Leu851fs*30 were located within the TPR domain, and variant p.Leu508* was located in the WD40 repeat domain. Both missense variants changed the hydrogen bonds (Figure 4a–d).

In the PDB database, the structure of the GEMIN5 protein has been fully resolved, and the complete amino acid sequence of the protein can be obtained. According to the prediction results, p.Leu902Gln results in the substitution of leucine (Leu) with glutamine (Gln). Figure 4a,b illustrates the impact of the Leu902Gln mutation on the structure and interactions with other amino acids. In the wild‐type protein, L902 forms hydrogen bonds with Y903 and R904. After the mutation, in addition to the two original hydrogen bonds, Q902 also forms hydrogen bonds with D898 and I906, respectively. Compared to the wild‐type protein, although the mutant protein shows a significantly increased number of hydrogen bonds, there is no apparent effect on the three‐dimensional structure. However, further experimental verification is required to determine whether this variant will alter the protein's activity.

The p.Gln971Glu results in the substitution of glutamine (Gln) with glutamic acid (Glu) in the amino acid sequence. Figure 4c,d illustrates the impact of the Gln971Glu mutation on the protein structure and interactions with other amino acids. In the wild‐type protein, Q971 forms hydrogen bonds with F968 and L972, respectively. After the mutation, in addition to the original two hydrogen bonds, E971 also forms a hydrogen bond with Q975. The mutant protein exhibits additional hydrogen bond formations compared to the wild‐type. The variation at this position can be clearly observed in the three‐dimensional structural prediction of the protein, affecting the original arrangement of some protein main chains and consequently altering the folding structure of certain protein regions.

### 3.4 Genotype‐phenotype correlation of *GEMIN5* variants

We analyzed the genotype‐phenotype relationship in all reported *GEMIN5* pathogenic variants with detailed clinical phenotypes. Previously, 45 kinds of *GEMIN5* variants in 48 patients have been reported in 5 published papers (Francisco‐Velilla et al., 2022; Ibrahim et al., 2023; Kour et al., 2021; Rajan et al., 2022; Saida et al., 2021). Including our 2 patients, a total of 49 variants in 50 patients were collected and analyzed. Clinical and molecular details of patients with epilepsy are listed in Table 1, and patients without epilepsy are listed in Table S1. Ten out of the 50 patients had epilepsy. Table 1 presents the genetic origin of each mutation site. We further analyzed the sub‐regional locations and found that half of those variants in 10 patients with epilepsy were located in the TPR‐like domain. The frequency of missense mutation is the highest. Two hotspot/recurrent variants, including p.Leu1367Pro (3 patients with epilepsy), located in RBS1, and p.lle988Phe (2 patients with epilepsy), located in TPR‐like domain, were observed (Tables 1 and 3). Seven of 10 patients presented with epilepsy within the first year of life, but detailed manifestations of seizures were not reported. Four patients were reported with a history of ASMs. Among them, our two patients with EIDEE had drug resistant epilepsy and still presented with seizures, whereas the other two were well controlled by CLB and LEV, respectively. All 10 patients with epilepsy had development delays (Table 1).

**TABLE 3:** Hotspot/recurrent variants of gem nuclear organelle–associated protein 5 (GEMIN5) gene.

Domain	Hotspot/Recurrent variants	Number of patients	Epilepsy Yes or No
WD40 repeat domain	p.Ser543Gly	2	No
	p.Pro594Arg	2	No
	p.Val611Met	3	No
	p.Gly683Asp	2	No
TPR‐like domain	p.Ala994Val	2	No
	p.Ala1007Thr	2	No
	p.Arg1016Cys	4	No
	p.His913Arg	3	No
	p.Ser1000Pro	4	No
	p.Asp1054_Ala1055 delins Glu	2	No
		1	Yes
	p.lle988Phe	2	No
		2	Yes
	p.Asp1019Glu	3	No
		1	Yes
RBS domain	p.Tyr1282His	1	No
		1	Yes
	p.Ser1311Leu fsX7	1	No
		1	Yes
	p.His1364Pro	2	No
	p.Leu1367Pro	3	No
		3	Yes

Abbreviation: TPR, tetratricopeptide repeat.

## 4 DISCUSSION

Brain abnormalities are the most common clinical manifestation of *GEMIN5*‐related neurodevelopmental disorders. Only one fifth of patients had seizures before 1 year old, mostly in the neonatal period (Francisco‐Velilla et al., 2022; Ibrahim et al., 2023; Kour et al., 2021; Rajan et al., 2022; Saida et al., 2021). Seizures in the neonatal period are linked to genetic and structural congenital abnormalities, potentially worsening the condition (Zuberi et al., 2022). Most *GEMIN5* gene mutation patients show cranial structural abnormalities, hinting at a link between seizures and disorder severity (Francisco‐Velilla et al., 2022; Ibrahim et al., 2023; Kour et al., 2021; Rajan et al., 2022; Saida et al., 2021). In our study, we found that both cases had neonatal epilepsy onset, burst suppression on EEG, and multiple seizure forms.

This study describes two females with novel biallelic *GEMIN5* variants inherited from asymptomatic parents who were predicted damaging or probably damaging using in silico tools. The two missense variants affect conserved residues observed in vertebrates. Patient symptoms overlap with reported *GEMIN5* mutation cases, suggesting *GEMIN5* variants as the cause (Ibrahim et al., 2023). Patients 1 and 2 share core *GEMIN5* variant features like cerebellar hypoplasia and developmental delay. Additionally, both have EIDEE, corpus callosum dysgenesis, and drug‐resistant epilepsy, new phenotypes. Patient 2 also has laryngeal chondrodysplasia, clubfoot, and hip dislocation. EIDEE prognosis varies, possibly evolving into different epilepsy syndromes (Guerrero Ruiz, 2022). Limited patient data hindered evolution summaries and prognostic comparisons, necessitating more *GEMIN5*‐related cases and long‐term follow‐up for comprehensive evaluation.

We searched databases and found 5 articles on *GEMIN5* variant patients, totaling 50 cases, with 47 showing cerebellar volume loss in MRI, a hallmark of NEDCAM syndrome (Francisco‐Velilla et al., 2022; Ibrahim et al., 2023; Kour et al., 2021; Rajan et al., 2022; Saida et al., 2021) (Table S1). Eight had epilepsy, but it was not a focus previously. Five had seizures in the first year, and our two had neonatal seizures, suggesting a link between EIDEE and *GEMIN5*. Comparing our patients with literature cases, we found overlapping phenotypes from *GEMIN5* mutations. Although the two missense variants were predicted to be damaging or probably damaging using in silico tools, we classified these variants as Variants of Unknown Significance because functional data are needed to conclude on their pathogenicity.

Variants p.Leu902Gln, p.Gln971Glu, p.Leu851fs30 in TPR‐like domain, and p.Leu508 in WD40 repeat domain. EIDEE likely links to TPR‐like variants, suggesting a genotype–phenotype correlation needing further study. Likely mechanism induced epilepsy may be that variants within TPR‐like domain fail to associate with native ribosomes, hampering its involvement in translation control and establishing a functional difference with the wild‐type protein (Francisco‐Velilla et al., 2022). In addition, a study in drosophila speculated that the absence of any one member of the SMN–GEMIN complex is sufficient to arrest its function in a nucleocentric pathway, which is critical for motor function in vivo (Borg & Cauchi, 2013). This reinforces the crucial role of the *GEMIN5* gene in the nervous system and the potential for its mutation to be a factor in the onset of neurological diseases. However, pathogenicity prediction via in silico tools needs confirmation through gene functional research.

Analysis of *GEMIN5* variant patients shows TPR‐like and RBS domain variants correlate with epilepsy (Table 3). A study showed that the variants in TPR‐like domain disrupt protein dimerization, whereas the RBS1 variants confer protein instability. Besides, mutants in these two domains have defects in their interaction with protein networks involved in translation and RNA‐driven pathways (Francisco‐Velilla et al., 2022; Piñeiro et al., 2015). GEMIN5 expression differences may impact clinical phenotypes and severity (Saida et al., 2021). RNA‐Seq analysis revealed that *SMN1*, *GEMIN3*, and *GEMIN5* are linked to a common set of genetic pathways, including the tp53 and ErbB pathways. All three genes facilitate regeneration by inhibiting the ErbB pathway, thereby allowing cell proliferation in the injured neuromasts (Pei et al., 2020). However, factors affecting clinical heterogeneity in GEMIN5 patients need further study.

## 5 CONCLUSION

In conclusion, we found novel biallelic variants of *GEMIN5* in two individuals with EIDEE, dysgenesis of the corpus callosum and drug‐resistant epilepsy, which expands the phenotypes and increases awareness of the association between *GEMIN5* variants and epilepsy. The genotypes and variant locations help explain the phenotypic heterogeneity of patients with *GEMIN5* variants.

## AUTHOR CONTRIBUTIONS

**Jing Zhang**: Writing—original draft. **Xinting Liu**: Formal analysis. **Gang Zhu**: Writing—review and editing. **Lin Wan**: Conceptualization. **Yan Liang**: Methodology. **Nannan Li**: Investigation. **Mingwei Huang**: Validation. **Guang Yang**: Writing—review and editing; funding acquisition; project administration; resources; supervision; data curation; software; visualization.

## CONFLICT OF INTEREST STATEMENT

All authors claim that there are no conflicts of interest.

## FUNDING INFORMATION

General Project of National Key Research and Development Program of China, Reference: 2023YFC2706405; Capital's Funds for Health Improvement and Research, Reference: 2024‐2‐5082; Beijing Natural Science Foundation, Reference: 7222187; Equipment Comprehensive Research Project of General Armament Department, Reference: 145BHQ090025000X; Key Project of Innovation Cultivation Fund of the Seventh Medical Center of Chinese PLA General Hospital, Reference: qzx‐2023‐1; Special Scientific Research Project of Military Family Planning, Reference: 22JSZ20.

### PEER REVIEW

The peer review history for this article is available at https://publons.com/publon/10.1002/brb3.3535.

## INFORMED CONSENT

The patients gave their informed consent for this report.

## Supporting information



Supporting Information

## Data Availability

The data that support the findings of this study are available in the Supporting Information section of this article or from the corresponding author upon reasonable request.
